# Do the placental barrier, parasite genotype and Toll-like receptor polymorphisms contribute to the course of primary infection with various *Toxoplasma gondii* genotypes in pregnant women?

**DOI:** 10.1007/s10096-013-2017-3

**Published:** 2013-11-30

**Authors:** W. Wujcicka, J. Wilczyński, D. Nowakowska

**Affiliations:** 1 Department of Fetal-Maternal Medicine and Gynecology, Polish Mother’s Memorial Hospital Research Institute in Lodz, 281/289 Rzgowska Street, Lodz, 93-338 Poland; 2 Department of Fetal-Maternal Medicine and Gynecology, Polish Mother’s Memorial Hospital Research Institute and Medical University of Lodz, 281/289 Rzgowska Street, Lodz, 93-338 Poland

## Abstract

*Toxoplasma gondii* has a highly clonal genetic structure classified into three major genetic types, I, II, and III, plus additional recombinant and atypical strains. In humans, type I and atypical strains usually associate with severe toxoplasmosis. Type II strains, predominantly identified in European countries and the United States, correlate with a differential course of toxoplasmosis. During pregnancy, the important protective role of the placenta against maternal–fetal *T. gondii* transmission has been reported. *T. gondii* preferentially colonizes extravillous trophoblasts as compared to syncytiotrophoblasts. The latter compartment was suggested to act as the real barrier to the fetal dissemination of *T. gondii*. Alterations in immune response to particular *T. gondii* strains were observed. Higher transcription levels of IP-10, IL-1β, IL-6, IL-10, IL-12 cytokines, and NF-κB translocation to the nucleus were more often documented for type II strains than type I strains. Since the induction of IL-12 during type II infection was Myd88-dependent, the involvement of Toll-like receptors (TLRs) in the immunity against these strains was suggested. Differential expression of TLRs depends on placental cell types and gestational age. The expression of TLR2 and TLR4 in the first trimester of pregnancy was reported only for villous cytotrophoblasts and extravillous trophoblasts, but not for syncytiotrophoblasts. The involvement of single-nucleotide polymorphisms (SNPs) in the *TLR* genes in infectious pathogenicity, including toxoplasmic retinochoroiditis, points at a possible involvement of *TLR* alterations in immunity against *T. gondii*. We conclude that studies on TLR contributions in the maternal–fetal transmission of particular parasite strains and congenital toxoplasmosis are warranted.

## *Toxoplasma gondii* genotypes contribute differentially to the course of toxoplasmosis

*Toxoplasma gondii* has a highly clonal genetic structure, with three major genetic types, I, II, and III, plus additional recombinant and atypical strains [1–4]. The three archetypic lineages were predominantly observed in North America and Europe, whereas more divergent genotypes were identified in French Guiana, Mexico, and Brazil [2, 5, 6].

Combined clinical and in vitro studies showed that outbreaks of toxoplasmosis presented with asymptomatic to symptomatic courses related to the genotypes involved. Differential virulence of *T. gondii* strains was observed for mice infected with parasites of particular lineages [7, 8]. Genotype I of *T. gondii* is most virulent for mice, inducing extensive parasite dissemination and sudden murine death. In contrast, genotype II causes non-fatal infection with much less tachyzoite dissemination [7]. However, strain-specific virulence varied between hosts, as was reported for mice and rats [8, 9]. Hence, it was suggested that *T. gondii* strains virulent for mice might not lead to a similar course of toxoplasmosis in humans.

Studies of humans with *T. gondii* mainly included cases of congenital toxoplasmosis and often originated in immune-deficient patients [7, 10–12]. Less frequent reports show symptomatic acquired toxoplasmosis in immune-competent patients [2, 5, 7]. *T. gondii* type II strains, identified predominantly in the populations of some European countries and the United States, were reported to generate congenital toxoplasmosis, including lethal infection, severe neuro-ocular involvement, isolated chorioretinitis, and/or latent toxoplasmosis [7]. However, type II strains were also isolated from benign or latent cases of toxoplasmosis, whereas type I and atypical strains are usually from severe cases [7, 13].

An atypical multi-locus *T. gondii* genotype identified from the amniotic fluid of a 29-year-old pregnant woman of Caucasian origin who was native to France was reported to cause severe congenital toxoplasmosis with bilateral ventricular enlargement and calcifications [1]. For at least 5 of 11 patients with laboratory-diagnosed toxoplasmosis living in Patam, a village near the French Guianan border, mouse inoculation or polymerase chain reaction (PCR) showed one atypical strain of *T. gondii* causing differential disease courses [5]. Eight immune-competent adults showed multi-visceral toxoplasmosis, leading to one death. One neonate and one fetus had lethal congenital toxoplasmosis and one child had symptomatic toxoplasmosis [5]. Hence, factors other than perceived strain virulence were suggested to influence the clinical presentation of toxoplasmosis in people living in an Amazonian rain forest [5].

The genotyping of 88 *T. gondii* isolates from immune-compromised patients in France showed a lack of significant differences in the distribution of parasite strains between patients diversified accordingly to cause of immunosuppression, site of infection, and outcome [14]. Hence, among immune-compromised patients, host factors were suggested as being co-correlated with toxoplasmosis development [14]. However, another study reported atypical *T. gondii* strains to have given rise to an outbreak of congenital toxoplasmosis and being responsible for more severe clinical courses of ocular toxoplasmosis in Brazilian children as compared to Europeans [15].

## *Toxoplasma gondii* genotypes differentially transmit through the placenta

Despite the fact that differential immune responses resulted from infection with various *T. gondii* strains, only a few studies reported that the parasite maternal–fetal transmission through the placenta might depend on strain variations. However, the protective role against congenital infections with *T. gondii* was reported for the placenta and its particular compartments.

In a BALB/c mice model, animals were inoculated 30 days before breeding with *T. gondii* ME49 (type II) or M7741 (type III) strains. The same mice were re-infected 12 and 15 days after pregnancy with other strains (M7741 or ME49, respectively) and did not develop congenital infections [16]. Mice which were primary infected during pregnancy had no fetal infection as well, although their placentas were *T. gondii*-positive. Hence, the placenta played an important protective role against the maternal–fetal transmission of *T. gondii* [16]. No fetal infection developed despite the fact that parasites could be detected in placentas, kidneys, spleens, livers, and hearts of the BALB/c mice [17]. A higher frequency of infected placentas was observed at later stages of pregnancy, which has been correlated with the phagocytic efficiency of the placental tissues, possibly related to the stage of pregnancy as well [18, 19].

Studies with first-trimester human placental explants described the role of two anatomical placental interfaces [the villous trophoblast (fetal cells) and the extravillous trophoblast (EVT, maternal cells)] in the maternal–fetal transmission of *T. gondii* [20]. Similar to other pathogens, *T. gondii* preferentially colonized the EVT and not the relatively resistant syncytiotrophoblasts (ST). The multi-parasite vacuoles were primarily identified within subsyncytial cytotrophoblasts, possibly representing transsyncytial *T. gondii* transmission. As only single parasites were observed in ST, this placental compartment was suggested to act as a barrier to the fetal dissemination of *T. gondii* [20]. The comparison between three *T. gondii* strains showed no significant differences in their capacity to infect placental interfaces; only a slightly slower replication rate of the type II strain was observed [20]. Another study reported that BeWo choriocarcinoma cells went into apoptosis after infection with type II rather than type I strains. In this case, host cell death versus parasite death might reflect differential strain abilities to infect the placenta with varying degrees of virulence [21].

An important protective function was reported for IFN-γ, the key regulator of anti-*T. gondii* immunity [19]. Pregnant transgenic IFN-γ knock-out (GKO) B6 (susceptible) and BALB/c (resistant) mice infected with *T. gondii* showed higher parasite numbers in the uterus and placenta than wild-type (WT) mice [19]. In addition, also, murine genetic susceptibility to parasite infection was shown to be a key factor. For instance, fetal infection was observed only in GKO B6 and not among GKO BALB/c, WT B6, and WT BALB/c mice [19].

## *Toxoplasma gondii* genotypes induce differential immune responses

In addition to the apparently differential virulence of various *T. gondii* genotypes, studies also showed altered immune responses against particular genotypes (Table 1) [22–25]. *T. gondii*-infected WT murine microglial cells showed variable kinetics of pro-inflammatory cytokine expression dependent on the parasite strain [22]. Higher and sustainable responses including elevated expression levels of IP-10 and IL-12b were observed in case of *T. gondii* II and III strains, but not for type I strains. The observed fluctuations in cytokine expression were time-dependent, and strain-dependent alterations of anti-apoptotic genes were minimal [22]. Oppositely, the infection of human neuroepithelioma cells with *T. gondii* type I strains caused bigger changes in the expression levels of a higher number of inflammation- and apoptosis-related genes than that observed for types II and III [25]. Macrophage immune responses to infection with *T. gondii* types I and II strains also showed a 2- to 3-fold elevated IL-12 gene expression [26]. Additionally, the induction of IL-12 expression after infection with type I strains did not involve Myd88 signaling, whereas this was clearly Myd88-dependent with type II strains.

**Table 1:** *Toxoplasma gondii* strain-dependent differences in immune response against parasites

Infected cell/animal	Immune response	Reference
Murine microglial cells	Increased expression of IP-10 after 2 h from infection with type I strain, significantly higher than with types II or III strains (*p* < 0.05). Higher sustained expression of IP-10 and IL-12b after infection with types II and III strain compared to type I strain (*p* < 0.05).	Glaser et al. (2011) [22]
Human neuroepithelioma cells	Altered expression level of a greater variety of genes associated with processes related to reproduction, response to stimulus, motility, metabolism, homeostasis, the central nervous system, inflammation, apoptosis, behavior, and transport observed after infection with type I strain than with types II or III strains (3.3 % of transcripts on array compared to only 0.4 % and 1.1 %, respectively).	Xiao et al. (2011) [25]
Murine bone marrow-derived macrophages (BMM)	Increased expression level of IL-12 cytokine approximately 2- to 3-fold higher after infection with type II strain compared to type I strain. Higher and longer lasting MAPK phosphorylation after type II compared to type I infection. Activation of p38 and ERK1/2 signaling through Myd88-dependent manner in case of type II strain infection and Myd88-independent way after type I infection.	Kim et al. (2006) [26]
Murine BMM	Nearly 200-fold higher production of IL-12 after type II than type I strain infection (*p* < 0.001). Induction of IL-12 expression dependent on Myd88 molecule in case of type II but not type I strain infection. NF-κB activation resulting in significantly higher NF-κB p65 nuclear localization in response to type II than type I strain infection (*p* < 0.005).	Robben et al. (2004) [27]
CD1 outbred mice	Higher expression level of IL-12p40 and IFN-γ after type II strain infection compared to type I infection.	Mordue and Sibley (2003) [31]
Murine BMM	Activation of NF-κB after infection with type II but not types I and III strains resulting from different activities of *T. gondii* GRA15 molecules.	Rosowski et al. (2011) [23]
Human foreskin fibroblasts	Massive production of pro-inflammatory cytokines early after infection with type II strain, but dampening expression of IL-12, IL-1β, and IL-6 after types I and III strains infection resulting from different activation of STAT3/6 signaling.	Saeij et al. (2007) [24]

The ME49 strain activated ERK1/2 and p38 MAPK in an Myd88-dependent manner and enhanced the expression of IL-12, which the RH strain failed to do [26]. Various signaling pathways involved in the immunity to *T. gondii* types I and II strains might affect the differential virulence of parasites [26]. Myd88-dependent IL-12 expression occurring after macrophage infection by type II strains but not type I strains was confirmed in another study [27]. Again, the involvement of Myd88 in the production of IL-12 suggested a role of Toll-like receptors (TLRs) in the immunity against *T. gondii* type II strains [27]. Types I and II strains of *T. gondii* also differentially influenced the activity of NF-κB, the transcription factor reported to play a key role in the induction of pro-inflammatory cytokine expression [28, 29]. NF-κB translocation to the nucleus was observed after the infection of mouse splenocytes or mouse bone marrow-derived macrophages (BMM) with the ME49 strain but not with the RH strain [27, 30]. The high IL-12 expression level was suggested to be specific for type II strain, as this elevated cytokine production was not inhibited by an earlier infection with a type I strain [27, 31]. ME49 induced also higher levels of IL-10, IL-1β, and IL-6 cytokines [27].

## TLRs expression levels differ within placental cells

So far, no study has been performed to analyze the role of TLRs, regulators of innate immunity, in the immune response to various *T. gondii* strains during pregnancy and in parasite transmission through the placenta. However, several studies reported the expression of TLRs in trophoblasts, decidual cells, and amniotic epithelium [32–34]. Among them, most studies reported variable TLR expression levels observed in trophoblasts [35–37]. For TLR4, elevated expression was observed in decidual cells as compared to interstitial trophoblasts. This suggested a possibly protective role of maternally derived cells [34]. Within the first-trimester placenta, the expression of TLR2 and TLR4 was observed only in villous cytotrophoblasts and EVTs, but not in ST [35]. This suggested that placental tissue and the fetus might be infected by microbes, which have passed through the breached TLR-negative ST [38].

Taking into account the above data, which confirm the preferential colonization of EVTs by *T. gondii*, an important role of placental TLRs in the immune response against *T. gondii* seems plausible. However, previous studies showed variable expression of other TLRs in different placental cells, which seemed to be related to the cell type as well as the stage of pregnancy [39–41]. In vitro studies using cultured placental cells showed that both cytotrophoblasts and ST cells express TLR2, TLR3, TLR4, TLR5, TLR6, and TLR9 [33]. Transcription of the genes encoding TLR1, TLR2, TLR3, and TLR4, but not TLR6 was reported for first-trimester trophoblasts [35]. Expression of TLR6 observed in third-trimester trophoblasts suggested its time-dependent regulation [33, 35].

Based on current data, we hypothesize that the gestational stage-dependent differences in transplacental transmission rates of *T. gondii* (ranging from 0 % in case of maternal infection acquired before gestation to 67 % when infection was acquired between weeks 31 and 34 of pregnancy) might be related to the stage-dependent activity of TLRs, which is to be confirmed by further studies [42, 43]. Simultaneously, the limited expression of TLRs on trophoblast cells at the early stages of pregnancy shows that these early cells are less able to deal with intrauterine infections than differentiated trophoblasts [38]. The TLR4 expression level was elevated at term as compared to first-trimester trophoblasts [44]. We suggest that description of the mechanisms of TLRs action after primary *T. gondii* infection of placental cells is needed. As differential replication rates were observed after the infection of trophoblasts with various parasite strains, investigation of the role of TLRs in the course of intrauterine infection with different *T. gondii* strains seems to be a challenge as well.

## SNPs in TLRs correlate with various infectious diseases

Single-nucleotide polymorphisms (SNPs) are common genetic alterations that may impact the expression levels of genes within which they are located. Studies of the involvement of *TLR* SNPs in the course of congenital toxoplasmosis seem necessary [29]. *TLR* polymorphisms were broadly investigated in the immune responses against various pathogens, including *Hepatitis C virus* (HCV), *Legionella pneumophila*, *Plasmodium falciparum*, *Mycobacterium leprae*, *Mycobacterium tuberculosis*, as well as *Human cytomegalovirus* (HCMV) [45–48]. Many studies demonstrated the involvement of different *TLR* SNPs in the course of inflammatory diseases and in the altered expression of TLR-dependent immune response genes [48–52].

The *TLR4* +896 allele was highly associated with post-meningitis hearing loss, especially in case of meningococcal meningitis (MM) [53]. SNPs located in the *TLR2*, *TLR4*, and *TLR9* genes were suggested as important modifications involved in immune response to BM and its clinical consequences [53]. In another study, the *TLR7* Leu11Gln polymorphism was associated with *Human immunodeficiency virus* (HIV) disease, including higher viral load and faster progression of advanced immunosuppression [54]. In case of HCV infection, important risk factors were, among others, *TLR7* IVS2 -151 G and *TLR8* -129 G polymorphisms [47]. The occurrence of CD14+ cells from subjects with *TLR7* IVS2 -151 A/*TLR8* -129 G (AG) haplotype with the expression of *TLR7* and *TLR8* was significantly lower than from individuals with GG and AC haplotypes [47]. The *TLR8* Met1Val polymorphism was reported to associate with both HIV and tuberculosis [55, 56]. Another study showed a slight effect of the *TLR8* Met1Val allele on TNF-α response [57]. 2258 G > A SNP residing in the *TLR2* gene was shown to correlate with severe phenotype in a subgroup of atopic dermatitis patients [48]. Carriers of the 2258 G > A allele showed higher risk for the development of atopy, increased levels of serum IgE and allergen-specific IgE antibodies [50]. The −16934 A > T SNP of the *TLR2* gene promoter region was related to decreased risk of allergic sensitization development, hay fever, and asthma in farmer’s children [58].

In case of the *TLR4* gene, the 1063 A > G and 1363 C > T SNPs were the most commonly studied. So far, these polymorphisms have been showed to associate with susceptibility to infectious diseases caused by Gram-negative bacteria, *Brucella* species, *Respiratory syncytial virus* (RSV), and *P. falciparum* [59–61]. Firstly, Arbour et al. reported 1063 A > G and 1363 C > T to be associated with weakened response toward inhaled lipopolysaccharide (LPS) [62]. Ducloux et al. showed a correlation of these two SNPs with rates of acute rejection and the occurrence of atherosclerotic events in kidney recipients [63]. According to the studies of Arbour et al. and Schwartz, it was shown that cells transfected with any of the *TLR4* haplotypes had decreased NF-κB activity compared with normal *TLR4* [62, 64–66]. A significant correlation was shown between 1063 A > G and 1363 C > T SNPs and RSV bronchiolitis in infants [61]. Few studies showed a correlation of *TLR9* polymorphisms with allergy or asthma [51, 67, 68]. Peixoto-Rangel et al. reported a correlation of the 1635 A > G SNP residing in the *TLR9* gene with toxoplasmic retinochoroiditis in Brazil [69]. In this population, ocular toxoplasmosis was associated with allele C at 1635 A > G (odds ratio = 7; 95 % confidence interval 1.6–30.8), which was at a frequency of 0.424, similar to that observed in European populations. The observed correlations suggested that direct interaction between *T. gondii* and TLR9 might trigger pro-inflammatory responses and, hence, lead to severe pathologies such as ocular disease associated with this infection in Brazil [69]. It seems important to describe the possible contribution of *TLR* SNPs to congenital toxoplasmosis [29].

## Concluding remarks

There is a clear need for a detailed description of mechanisms of congenital toxoplasmosis development. During the maternal–fetal transmission of *Toxoplasma gondii*, preferential colonization of extravillous trophoblasts (EVTs) by the parasite was observed. Several studies showed different expression levels of Toll-like receptors (TLRs), dependent on placental cell type and stage of pregnancy. In case of TLR2 and TLR4, the expression was identified in first-trimester villous cytotrophoblasts and EVTs, but not in syncytiotrophoblasts (STs). Hence, an important role of TLRs against the development of fetal *T. gondii* infection seems plausible. SNPs located in various *TLR* genes associate with differential infectious diseases, including toxoplasmic retinochoroiditis. As a differential immune response has been reported in correlation with particular *T. gondii* genotypes, the contribution of TLRs in the course of congenital infection with various *T. gondii* strains seems to be likely as well. We suggest further studies of TLRs in the maternal–fetal transmission of particular parasite strains and congenital toxoplasmosis as being extremely important.
